# Deep Local Analysis deconstructs protein–protein interfaces and accurately estimates binding affinity changes upon mutation

**DOI:** 10.1093/bioinformatics/btad231

**Published:** 2023-06-30

**Authors:** Yasser Mohseni Behbahani, Elodie Laine, Alessandra Carbone

**Affiliations:** Laboratory of Computational and Quantitative Biology (LCQB), UMR 7238, Sorbonne Université, CNRS, IBPS, Paris 75005, France; Laboratory of Computational and Quantitative Biology (LCQB), UMR 7238, Sorbonne Université, CNRS, IBPS, Paris 75005, France; Laboratory of Computational and Quantitative Biology (LCQB), UMR 7238, Sorbonne Université, CNRS, IBPS, Paris 75005, France

## Abstract

**Motivation:**

The spectacular recent advances in protein and protein complex structure prediction hold promise for reconstructing interactomes at large-scale and residue resolution. Beyond determining the 3D arrangement of interacting partners, modeling approaches should be able to unravel the impact of sequence variations on the strength of the association.

**Results:**

In this work, we report on Deep Local Analysis, a novel and efficient deep learning framework that relies on a strikingly simple deconstruction of protein interfaces into small locally oriented residue-centered cubes and on 3D convolutions recognizing patterns within cubes. Merely based on the two cubes associated with the wild-type and the mutant residues, DLA accurately estimates the binding affinity change for the associated complexes. It achieves a Pearson correlation coefficient of 0.735 on about 400 mutations on unseen complexes. Its generalization capability on blind datasets of complexes is higher than the state-of-the-art methods. We show that taking into account the evolutionary constraints on residues contributes to predictions. We also discuss the influence of conformational variability on performance. Beyond the predictive power on the effects of mutations, DLA is a general framework for transferring the knowledge gained from the available non-redundant set of complex protein structures to various tasks. For instance, given a single partially masked cube, it recovers the identity and physicochemical class of the central residue. Given an ensemble of cubes representing an interface, it predicts the function of the complex.

**Availability and implementation:**

Source code and models are available at http://gitlab.lcqb.upmc.fr/DLA/DLA.git.

## 1 Introduction

The ever-growing number of sequenced individual genomes and the possibility of obtaining high-resolution 3D structural coverage of the corresponding proteomes (Jumper et al. 2021; Mirdita et al. 2021) open up exciting avenues for personalized medicine. Assessing the impact of sequence variations, particularly missense mutations, between individuals on how proteins interact with each other can shed light on disease susceptibility and severity (Creixell et al. 2015; Jubb et al. 2017) and help decipher gene–disease–drug associations for developing therapeutic treatments (Hao et al. 2012; Piñero et al. 2015; Tang et al. 2020). Of particular interest are the surface regions of proteins directly involved in the interactions, as this is where most disease-related missense mutations occur (David et al. 2012; Gonzalez and Kann 2012; David and Sternberg 2015; Xiong et al. 2022). At the same time, rapid advances in deep learning techniques for biology, especially for biomolecules, are creating opportunities to revisit the way we look at protein complexes and represent them. The impact of a mutation on the strength of the association between two protein partners can be measured by the difference in binding free energy where $\Delta G_{\text{Bind}}^{\text{MU}}$ and $\Delta G_{\text{Bind}}^{\text{WT}}$ are the binding free energies, or binding affinities, of the mutated and wild-type complexes, respectively. Significant efforts have been expended over the past decade to produce, collect and curate binding affinity measurements for wild-type and mutated complexes (Supplementary Table S1) (Moal and Fernández-Recio 2012; Vreven et al. 2015; Sirin et al. 2016; Jemimah et al. 2017; Liu et al. 2018; Jankauskaitė et al. 2019). Nevertheless, the handful of experimental techniques yielding accurate estimates of $\Delta G_{\text{Bind}}$ remain laborious, expensive, and time-consuming (Vangone and Bonvin 2015). To overcome this limitation, several efficient computational methods have been developed (Supplementary Table S2) (Guerois et al. 2002; Pires and Ascher, 2016; Xiong et al. 2017; Barlow et al. 2018; Geng et al. 2019; Rodrigues et al. 2019, 2021; Liu et al. 2020, 2022; Wang et al. 2020; Zhang et al. 2020; Zhou et al. 2020). Most of them exploit local environments around the mutation site to directly predict $\Delta \Delta G_{\text{Bind}}$ values. The advantage of this strategy is 2-fold. First, it avoids the accumulation of errors on the $\Delta G_{\text{Bind}}^{\text{WT}}$ and $\Delta G_{\text{Bind}}^{\text{MU}}$ quantities that would result in large approximations in $\Delta \Delta G_{\text{Bind}}$. Second, it avoids the unnecessary calculation of properties not modified by the mutation, e.g. the chemical composition of the noninteracting surface and the 3D geometry of the interface contact distribution. Indeed, these properties, while contributing strongly to the binding affinity (Vangone and Bonvin 2015; Raucci et al. 2018), are not, or only slightly, sensitive to point mutations located at the interface. The state-of-the-art methods sometimes achieve very high prediction accuracy when evaluated using 10-fold cross validation. However, their ability to generalize to diverse complexes and across different databases can be improved (Geng et al. 2019).


(1) $$\Delta \Delta G_{\text{Bind}}=\Delta G_{\text{Bind}}^{\text{MU}}-\Delta G_{\text{Bind}}^{\text{WT}},$$

Representation learning powered by deep neural networks has opened up opportunities to develop all-purpose models transferring knowledge across systems and tasks. After a major breakthrough in natural language processing (Vaswani et al. 2017; Devlin et al. 2019), the concept has been transferred to proteins through protein language models (pLMs) (Heinzinger et al. 2019; Bepler and Berger 2021; Elnaggar et al. 2021; Rives et al. 2021). pLMs learn the fundamental properties of natural protein diversity by reconstructing some masked or the next amino acid(s), given their sequence context, at scale. They exhibit exciting potential for a broad range of protein-related problems. Beyond sequence information, self-supervised learning-based approaches have leveraged the protein and protein complex 3D structures available in the Protein Data Bank (PDB) (Berman et al. 2002) for fixed-backbone protein design (Anand et al. 2022; Dauparas et al. 2022; Hsu et al. 2022), for predicting protein stability (Blaabjerg et al. 2022; Zhang et al. 2022), and for assessing the impact of mutations on protein–protein interactions (Liu et al. 2020). In particular, in Liu et al. (2020), a graph neural network is trained to reconstruct disturbed wild-type and mutated complex structures represented as graphs. A gradient-boosting trees algorithm then exploits the learned representations to predict mutation-induced $\Delta \Delta G_{\text{Bind}}$ values. Although this approach showed promising results, it sequentially employs two different machine learning components trained independently, limiting its versatility and applicability to other tasks.

**Figure 1.**
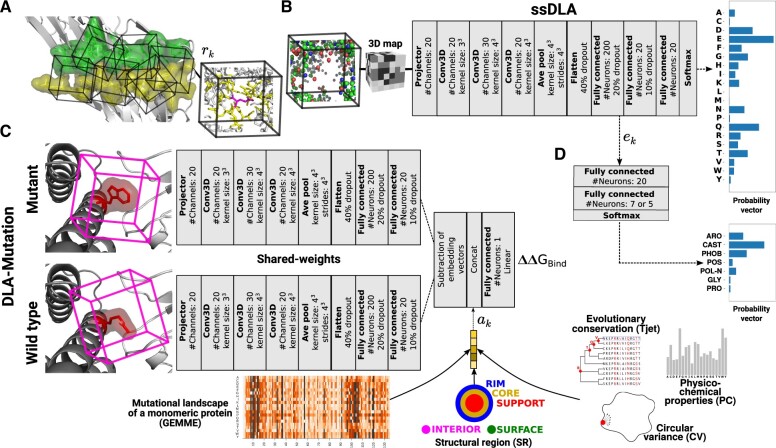
DLA data representations and architectures. (A) A representation of a protein interface (green and yellow residues from each partner) as an ensemble of cubes (*I_C_*). Each cube ($r_{k}\in I_{C}$) is centered and oriented around an interfacial residue. In the example cube on the right, the atoms displayed in yellow and magenta sticks are enclosed in a 5-Å-radius sphere centered on a randomly chosen atom from the central residue. (B) Architecture of the self-supervised model, named ssDLA. The input cube is the same as in panel (A). The atoms that were in yellow and magenta sticks are now replaced by an empty space. Carbon atoms are colored in green, oxygen in red, nitrogen in blue, and sulfur in yellow. The training task is to recover the identity of the residue lying at the center of the partially masked input cube. (C) Siamese architecture of the supervised model DLA-mutation predicting mutation-induced binding affinity changes. The two parallel branches with shared weights apply 3D convolutions to the local 3D environments around the wild-type and mutated residues and compute two embedding vectors. Auxiliary features are concatenated to the vector resulting from subtracting these two embedding vectors. (D) Two-layer dense classifier taking as input the embedding vectors computed by the pre-trained ssDLA (panel B) and outputting a probability vector whose dimension is the number of classes.

Here, we report on ‘Deep Local Analysis (DLA)-mutation’, the first deep learning architecture estimating mutation-induced $\Delta \Delta G_{\text{Bind}}$ from patterns in local interfacial 3D environments learnt through self-supervision (Fig. 1). It relies on a representation of protein interfaces as sets of locally oriented cubes we previously introduced in Mohseni Behbahani et al. (2022) and Pagès et al. (2019) (Fig. 1A). In this work, we leveraged this representation through self-supervised learning (Fig. 1B) and combined it with supervised learning of $\Delta \Delta G_{\text{bind}}$ exploiting both structural and evolutionary information (Fig. 1C). DLA-mutation only takes as input two cubes, corresponding to the environments around the wild-type and mutated residues, respectively, and directly estimates $\Delta \Delta G_{\text{bind}}$. Beyond prediction, we used the learned representations to investigate the extent to which the environment of an interfacial residue is specific to its type and physicochemical properties (Fig. 1D). DLA-mutation code and models are freely available to the community at http://gitlab.lcqb.upmc.fr/DLA/DLA.git.

## 2 Methods

### 2.1 Protein–protein interface representation

We represent a protein–protein interface as a set of locally oriented cubic volumetric maps centered around each interfacial residue (Fig. 1A). The local atomic coordinates of the input structure are first transformed to a density function, where each atom is one-hot encoded in a vector of 167 dimensions (Pagès et al. 2019). Then, the density is projected on a 3D grid comprising 24×24×24 voxels of side 0.8 Å. The map is oriented by defining a local frame based on the common chemical scaffold of amino acid residues in proteins (Pagès et al. 2019) (see Supplementary Data for more details). This representation is invariant to the global orientation of the structure while preserving information about the atoms and residues relative orientations.

For the self-supervised representation learning, we trained DLA to recognize which amino acid would fit in a given local 3D environment extracted from a protein–protein interface. Our aim in doing so is to capture intrinsic patterns underlying the atomic arrangements found in local interfacial regions. Formally, the machine predicts the probability P(y|env) of the amino acid type *y*, for y∈{A,C,D,…,W,Y}, conditioned on the interfacial local chemical environment env given as input. In practice, we process the input cube before giving it to DLA by masking a sphere of radius *r_c_*Å centered on an atom from the central residue (Supplementary Fig. S1 and Fig. 1A). Masking a fixed volume prevents introducing amino acid-specific shape or size biases. We experimented with different values of *r_c_* (3 and 5 Å) and different choices for the atom ($C_{\alpha },\;C_{\beta }$, random). We found that a sphere of radius of 5 Å with a randomly chosen center yielded both good performance and expressive embedding vectors.

For the supervised prediction of $\Delta \Delta G_{\text{Bind}}$, we combined the embeddings of the volumetric maps with five pre-computed auxiliary features (Fig. 1C), among which four describe the wild-type residue, namely its conservation level *T*_JET_ determined by the Joint Evolutionary Trees method (Engelen et al. 2009), its physicochemical properties to be found at interfaces (PC), its protruding character, as measured by its circular variance (CV) (Mezei 2003; Ceres et al. 2012), and the structural region (SR) where it is located: interior (INT), non-interacting surface (SUR), or, if it is part of the interface, support (S or SUP), core (C or COR), or rim (R or RIM) as defined in Levy (2010). We previously demonstrated the usefulness of these properties for predicting and analyzing protein interfaces with other macromolecules (protein, DNA/RNA) (Laine and Carbone, 2015; Raucci et al. 2018; Corsi et al. 2020; Mohseni Behbahani et al. 2022). The fifth feature is a numerical score computed by GEMME (Laine et al. 2019) that reflects the impact of the point mutation on the function of the protein chain where it occurs, considered as a monomer. GEMME combines the conservation levels *T*_JET_ with amino acid frequencies and the minimum evolutionary distance between the protein sequence and an homologous protein displaying the mutation. See Supplementary Data for more details.

### 2.2 DLA architectures

Our DLA framework is relatively simple, generic, and versatile. Its main core architecture comprises a projector, three 3D convolutional layers, an average pooling layer, and a fully connected subnetwork (Fig. 1B and C). The purpose of the projector is to reduce the dimension of each input cube voxel’s feature vector from 167 to 20. We apply batch normalization after each 3D convolution. The average pooling layer exploits scale separability by preserving essential information of the input during coarsening of the underlying grid. To avoid overfitting, we applied 40%, 20%, and 10% dropout regularization to the input, the first, and the second layers, respectively, of the fully connected subnetwork.

For the self-supervised task (Fig. 1B), the fully connected subnetwork contains three successive layers (sizes 200, 20, and 20) and the last activation function (Softmax) outputs a probability vector of size 20 representing the 20 amino acids. The categorical cross-entropy loss function measures the difference between the probability distribution of the predicted output and a one-hot vector encoding the true amino acid type of the central residue. We refer to this version of DLA to build the pre-trained model as ‘self supervised-DLA’ or ‘ssDLA’.

For the supervised $\Delta \Delta G_{\text{Bind}}$ prediction (Fig. 1C), we used the core DLA framework to build a Siamese architecture constituted by two branches with shared weights. The network processes two input cubes corresponding to the wild-type and mutated residues. The average pooling layer is followed by two fully connected layers of size 200 and 20, respectively, within each branch. We then merge the two branches by subtracting the computed embedding vector and concatenate the auxiliary features (described above) to the resulting vector. The last fully connected layer displays a linear activation function and outputs one value. The loss is the mean squared error. We refer to this architecture as ‘DLA-mutation’.

## 3 Databases

We computed the ground-truth $\Delta \Delta G_{\text{Bind}}$ values from SKEMPI v2.0 (Jankauskaitė et al. 2019), the most complete source for experimentally measured binding affinities of wild-type and mutated protein complexes. We restricted our experiments to the data produced by the most reliable experimental techniques, namely Isothermal Titration Calorimetry, Surface Plasmon Resonance, Spectroscopy, Fluorescence, and Stopped-Flow Fluorimetry, as done in Vangone and Bonvin (2015). We selected a subset of 2003 mutations associated with 142 complexes, referred to as ‘S2003’ in the following. To provide ssDLA and DLA-mutation with input protein–protein complex 3D structures, we created and processed two databases, namely ‘PDBInter’ and ‘S2003-3D’. PDBInter is a non-redundant set of 5055 experimental structures curated from the PDB. S2003-3D contains 3D models generated using the ‘backrub’ protocol implemented in Rosetta (Smith and Kortemme 2008). We refer to each generated conformation as a backrub model. We generated 30 backrub models for each wild-type or mutated complex. This amount was shown to be sufficient for estimating free energies in Barlow et al. (2018). See Supplementary Data for more details.

### 3.1 Training and evaluation of ssDLA and DLA-mutation

We trained and validated ssDLA on the PDBInter database. The protein complexes in the train set do not share any family level similarity with the 142 complexes from S2003, according to the SCOPe hierarchy (Fox et al. 2014; Chandonia et al. 2022). We generated 247 662 input samples (interfacial cubes) from the train set and 34 174 from the validation set. Amino acids are not equally distributed in these sets; leucine is the most frequent one, while cysteine is the rarest (Supplementary Fig. S2). To compensate for such imbalance and with the aim of penalizing more those errors that are made for the less frequent amino acids, we assigned a weight to the loss of each amino acid type that is inversely proportional to its frequency of occurrence (Supplementary Table S3). We trained ssDLA for 50 epochs with the Adam optimizer in TensorFlow at a learning rate of 0.0001 (Supplementary Fig. S3A). We explored different hyperparameter values by varying the learning rate, applying different normalization schemes, changing the compensation weights, etc. We retained the hyperparameters leading to the best performance on the validation set. The trained ssDLA model extracts ‘embedding vector’ *e_k_* of size 200 (Fig. 1B) for a given cube.

We used S2003 to train and test DLA-mutation. We set the learning rate at 0.001 and we initialized the weights of the network with those of the pre-trained ssDLA model. We first evaluated DLA-Mutation through a 10-fold cross validation performed at the mutation level. This evaluation procedure, which is widely used in the literature (Geng et al. 2019; Rodrigues et al. 2019, 2021; Wang et al. 2020; Zhou et al. 2020; Liu et al. 2022), considers each sample independently when splitting the data between train and test sets (‘mutation-based’ split). However, this assumption is problematic since the same complex or even the same wild-type residue may be seen during both the training and the testing phases. These cases are expected to be ‘easy’ to deal with. For a more challenging and realistic assessment, we held out 32 complexes displaying 391 mutations for the testing phase, and trained DLA-mutation on the rest of the dataset (‘complex-based’ split).

For the comparison with iSEE, we used the same train and test procedure as that reported in Geng et al. (2019) (Supplementary Table S2), using the wild-type and mutant 3D models produced by HADDOCK (van Zundert et al. 2016) and available from Geng et al. (2019). For the comparison with the other predictors, we defined the test set from the intersection between S2003 and the benchmark set used in Geng et al. (2019). It amounted to 112 mutations from 17 complexes. We defined a new training set comprising 945 mutations from S2003 coming from complexes sharing less than 30% sequence identity with those from this test set. In the case of GraphPPI (Liu et al. 2020), TopNetTree (Wang et al. 2020), and Hom-ML-V2 (Liu et al. 2022), the comparison remains qualitative due to the lack of complete readily available software packages and already trained models.

### 3.2 Mapping the embeddings to residue and interface properties

We trained a fully connected network composed of only one hidden layer of size 20 to map the embeddings computed by ssDLA to residue- and interface-based properties. The input layer is of size 200 and the Softmax activation function of the output layer computes a probability vector whose size is the number of classes. We used categorical cross-entropy as the loss function. In the first experiment, we mapped an input embedding vector (*e_k_*, size 200, see Fig. 1D), representing a local 3D interfacial environment, to an output amino acid physicochemical class, among the seven defined in Laine et al. (2019) (Supplementary Table S4). We directly gave the embedding computed by ssDLA for a given input cube to the classifier. In the second experiment, we mapped an input embedding averaged over an entire interface to an output interaction functional class, among antibody–antigen (AB/AG), protease–inhibitor (Pr/PI), and T-cell receptor—major histocompatibility complex (TCR/pMHC), as annotated in the SKEMPI v2.0. For training purposes, we redundancy reduced the set of 142 complexes from S2003 based on a 30% sequence identity cutoff. See Supplementary Data for details.

## 4 Results

The DLA framework deconstructs a protein–protein interface to predict mutation-induced changes in binding affinity and solve residue- or interface-based downstream tasks (Fig. 1). It extracts embedding vectors from locally oriented cubes surrounding wild-type or mutant interfacial residues and combines them with auxiliary features, including SRs or evolutionary information.

### 4.1 Can an interfacial residue be learnt from its environment?

**Figure 2.**
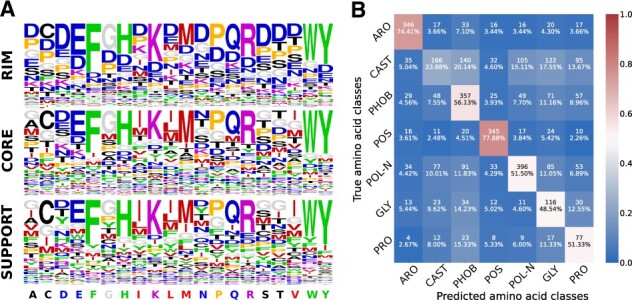
Performance of ssDLA model. (A) The predictive power of ssDLA model is evaluated on the validation set of ‘PDBInter’. The three logos represent the propensities of each amino acid to be predicted (having maximum score in the output layer), depending on the true amino acid (*x*-axis) and on its SR (see Section 2). Amino acids are colored based on seven similarity classes: ARO (F, W, Y, H) in green, CAST (C, A, S, T) in black, PHOB (I, L, M, V) in red, POS (K, R) in purple, POL-N (N, Q, D, E) in blue, GLY (G) in gray and PRO (P) in orange (see Section 2). (B) Confusion matrix for the prediction of the seven amino acid classes using embedding vectors generated by ssDLA. The percentage values and the colors indicate recall. The model is trained and tested on the interfacial residues of X-ray crystal structures of S2003. See Supplementary Fig. S5 for the performance of ssDLA when only four channels corresponding to the four amino acid-independent chemical elements (O, C, N, and S) are considered to define the cubic volumetric maps.

ssDLA was trained in a self-supervised way on experimental complex structures (PDBInter database, see Section 2). Its ability to recover the identity of the central residue in the input cube can inform us about the extent to which an interfacial residue’s 3D environment is specific to its amino acid type or physicochemical properties. To investigate this possibility, we analyzed the probability vectors computed by ssDLA when given a partially masked cube as input (Fig. 2A).

To avoid any amino acid-specific bias, we masked a volume of constant shape and size, namely a sphere of radius 5 Å, in all training samples (see Section 2 and Supplementary Fig. S1). ssDLA successfully and consistently recognized the amino acids containing an aromatic ring (F, Y, W, H) and most of the charged and polar ones (E, K, R, and to a lesser extent Q and D), as well as methionine (M), cysteine (C), glycine (G), and proline (P), whatever their SR (Fig. 2A). In contrast, the location of alanine (A), isoleucine (I), and leucine (L) influenced their detection. While they were ranked in the top 3 in support and core, they were almost never recognized in the rim. Inversely, the polar asparagine (N) was recognized when located in the rim or the core, but not the support. The model often confused the hydroxyl-containing serine (S) and threonine (T) on the one hand, and the hydrophobic I and L on the other hand.

These tendencies cannot be deduced from the relative frequencies of occurrence of the different amino acids in the three interface SRs (Supplementary Fig. S2). For instance, ssDLA behaves very differently with N and Q (Fig. 2A), although they display the same relative abundances and the same SRs preferences (Supplementary Fig. S2). Hence, the poor recovery rate for N suggests that the environments for this amino acid are more ambiguous or diverse than those observed for Q. Likewise, ssDLA tendency to over-populate the rim with aspartate (D) does not reflect its overwhelming presence in this region. We hypothesize that D serves as a ≪ bin ≫ class predicted when the environment is underdetermined. Such underdetermination or ambiguity is more likely to happen in the rim, where the residues are more exposed and thus the cube contains more empty space. A previous study reported different trends for a similar task and similar data representation (Anand et al. 2022). In particular, it could identify G and P with very high success, whereas it confused F, Y, and W. These results may reflect a bias toward recognizing amino acid-specific sizes and shapes, due to masking only the side chain of the central residue. Moreover, the model was trained and evaluated on monomeric proteins.

The spherical mask of radius 5 Å may not always cover the whole central residue, raising the question of whether the network relies on the amino acid-specific types of the remaining atoms in such cases. To test this, we removed any amino acid-specific information by reducing the 167 feature channels encoding the atom types to 4, corresponding to the four chemical elements C, N, O, and S. Even with four channels, ssDLA successfully recognized and distinguished the large aromatic amino acids F, W, and Y, as well as the long positively, charged R and K, whatever the SR (Supplementary Fig. S5). We also slightly lowered the weight of D in the calculation of the loss during training (Supplementary Table S3). This small change shifted the tendency of ssDLA to predict D for E, especially in the rim region (Supplementary Fig. S5). Such instability highlights the under-determination of the environments in this region.

### 4.2 DLA-mutation accurately predicts $\Delta \Delta G_{\text{Bind}}$

To build the DLA-mutation model, we fine-tuned the weights of the pre-trained ssDLA model (Fig. 1B) to predict $\Delta \Delta G_{\text{Bind}}$ values in a supervised fashion (Fig. 1C). Starting from a set of experimental structures of wild-type complexes, we generated 3D conformations for wild-type and mutated forms using the ‘backrub’ protocol implemented in Rosetta and we used them to train and test DLA-mutation (S2003-3D database, see Section 2 and Supplementary Fig. S6). For each mutation, we combined information coming from the local 3D environments of the wild-type and mutant residues extracted from the corresponding modelled 3D complexes with additional structural and evolutionary information.

**Figure 3.**
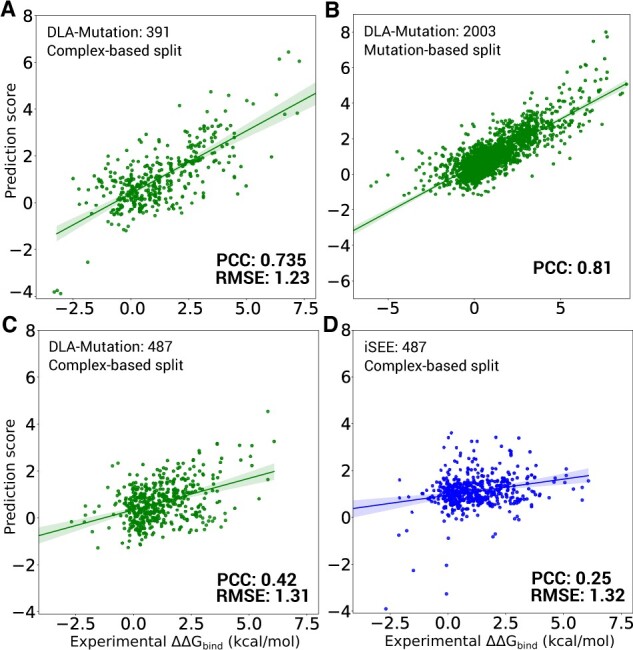
Performance of DLA-mutation and comparison with iSEE. (A and B) DLA-mutation scores versus experimental $\Delta \Delta G_{\text{Bind}}$ values on S2003 dataset. The models rely on fine-tuned weights, starting from those of the pre-trained ssDLA, and exploits all auxiliary features (see Section 2). (A) Test set of 391 mutations coming from 32 complexes that were not seen during training and were randomly selected from the S2003 dataset. (B) Mutation-based 10-fold cross validation procedure over all 2003 mutations. (C and D) DLA-mutation (green) and iSEE (blue) scores versus experimental $\Delta \Delta G_{\text{Bind}}$ values for the test set of 487 mutations from 56 complexes (S487 dataset). The input 3D models and training and evaluation procedure were directly taken from (Geng et al. 2019). (C) DLA-mutation (D) iSEE.

DLA-mutation achieved an overall very good agreement with $\Delta \Delta G_{\text{Bind}}$ experimental measurements (Fig. 3). It reached a Pearson correlation coefficient (PCC) of 0.735 and a root mean-squared error (RMSE) of 1.23 kcal/mol on 391 mutations coming from 32 complexes (Fig. 3A and Table 1). All testing complexes were different from the complexes seen during training (see Section 2). Hence, this result emphasizes DLA-mutation’s high generalization capability to unseen complexes.

**Table 1.** Different experimental setups for $\Delta \Delta G_{\text{bind}}$ prediction with DLA-mutation.

Train/test	No. (mutations)		Weight	Auxiliary	PCC	RMSE
split level	Train	Test	initialization	features		($\frac{\text{kcal}}{\text{mol}}$)
Mutation	2003	–	Pre-training	All	0.812	–

Complex	1612	391	Pre-training	SR	0.686	1.31
				SR-Tjet	0.712	1.32
				SR-GEMME	0.726	1.27
				All	0.735	1.23
	
			Random	SR	0.602	1.44
				All	0.657	1.37

Complex and	945	112	Pre-training	All	0.481	1.14
<30% seq. id.						

Complex	1102^a^	487^a^	Pre-training	All	0.423	1.31

a The input 3D models, generated by HADDOCK (van Zundert et al. 2016), were taken from Geng et al. (2019).

### 4.3 Evolutionary information and pre-training matter

As auxiliary features, we used the SR of the wild-type residue (Fig. 1C, SR), as well as other geometrical (CV), physicochemical (PC), and evolutionary (GEMME, *T*_JET_) descriptors. We performed an ablation study to assess the contribution of these descriptors and of the pre-training step (Supplementary Fig. S4 and Table 1).

In the baseline configuration, we used only the structure-based SR auxiliary feature. The latter informs the model about the location of the input cube’s central residue on the interface. We have previously shown that the SR information contributes significantly to the performance of the DLA framework (Mohseni Behbahani et al. 2022). Hence, the baseline version of the model does not include any sequence-based auxiliary feature. In addition to this, we also considered evolutionary information, using either GEMME scores (SR-GEMME) or *T*_JET_ conservation levels (SR-*T*_JET_). We found that the wild-type residue’s buriedness (CV) and interface propensity (PC) contributed very little to the accuracy of the predictions (Supplementary Fig. S4A–D and Table 1, compare All with SR-*T*_JET_ and SR-GEMME). Removing them is essentially harmless. In contrast, evolutionary information does significantly contribute to the model’s performance, as attested by the rather low PCC (0.648) obtained when using only SR (Supplementary Fig. S4B and Table 1). By design, the mutation-specific GEMME score is correlated to the position-specific conservation level *T*_JET_ (Laine et al. 2019), and thus the two descriptors are redundant to some extent. Nevertheless, we observed that the former was more informative than the latter (Supplementary Fig. S4, compare panels C and D). Finally, pre-training the architecture through self-supervision with ssDLA clearly improved the predictions (Supplementary Fig. S4, compare panels A and B with panels E and F, and Table 1). The gain in PCC is of 0.08 compared with initializing DLA-mutation weights randomly.

### 4.4 Comparison with state-of-the-art predictors

We considered three recent deep learning-based approaches, namely GraphPPI (Liu et al. 2020), TopNetTree (Wang et al. 2020), and Hom-ML-V2 (Liu et al. 2022). The reported performance for GraphPPI using leave-one-structure-out cross validation is similar to those we obtained for DLA-mutation (Supplementary Table S2). TopNetTree achieves a much lower PCC of 0.53 on the blind test. Both TopNetTree and Hom-ML-V2 were mainly evaluated using mutation-based cross validation (Supplementary Table S2, results marked with *). Such evaluation likely leads to overly optimistic estimates, since the same complex, or even the same mutation site, can be shared between the train and test sets. The PCC reported are as high as 0.85 on a set of 1131 mutations exclusively coming from the SKEMPI v1 dataset (Supplementary Table S2). By comparison, we obtained a PCC of 0.81 over 2003 single-point mutations following a mutation-based 10-fold cross validation (Fig. 3B). The slightly lower performance of DLA-mutation may come from the fact that by using SKEMPI v2.0 (which includes v1.0) we cover a larger number of complexes and experimental techniques for the estimation of binding affinity (see Section 2).

To provide a more controlled and precise comparison with the competitive methods and to further assess DLA-mutation generalization capabilities, we performed two experiments. In the first one, we reproduced exactly the train and test procedure described in Geng et al. (2019) for assessing iSEE and we applied it to DLA-mutation (Fig. 3C and D). iSEE is a recently developed machine learning-based method that, similarly to DLA-mutation, directly estimates $\Delta \Delta G_{\text{Bind}}$ values exploiting structural information coming from the wild-type and mutant complex 3D structures, as well as evolutionary information. We used HADDOCK-generated 3D models available from Geng et al. (2019) as input. The comparison is directly made to the iSEE results reported in Geng et al. (2019). We found that DLA-mutation generalized better than iSEE from SKEMPI version 1 to version 2 (Fig. 3C and D). Specifically, when DLA-mutation is trained on SKEMPI v1.0, it reached a PCC of 0.423 on 487 mutations coming from 56 unseen complexes from SKEMPI v2.0 (Fig. 3C). The correlation obtained with iSEE was much lower, around 0.25 (Fig. 3D). The baseline version of DLA-mutation, which relies only on structural information, still compares favorably to iSEE (Supplementary Fig. S7).

**Figure 4.**
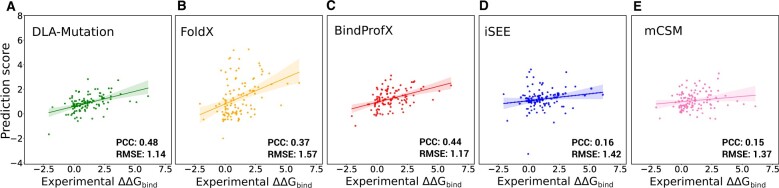
Comparison between DLA-mutation and other $\Delta \Delta G_{\text{Bind}}$ predictors. We report values for 112 mutations coming from 17 protein complexes not seen during the training or optimization of any of the predictors. (A) DLA-mutation was trained on 945 mutations from S2003 coming from complexes sharing less than 30% sequence identity with those from this test set. We used fine-tuning of the weights and all auxiliary features. (B and E) The scores reported for FoldX (B), BindProfX (C), iSEE (D), and mCSM (E) were taken directly from Geng et al. (2019).

In the second experiment, we extended the comparison to three other $\Delta \Delta G_{\text{Bind}}$ predictors, namely mCSM (Pires et al. 2014), FoldX (Guerois et al. 2002), and BindProfX (Xiong et al. 2017) (Fig. 4). mCSM directly estimates $\Delta \Delta G_{\text{Bind}}$ values by exploiting the 3D structure of the wild-type complex and descriptors of the substituting amino acid within a machine learning framework. FoldX estimates free energies of binding $\Delta G_{\text{Bind}}$ of the wild-type and mutant complexes using a physics-based energy function and then computes their difference. BindProfX combines FoldX with evolutionary interface profiles built from structural homologs. Without relying on machine learning, it achieves a good correlation with the experimental data from SKEMPI v1.0 (Supplementary Table S2). We found that DLA-mutation outperforms all of the predictors on a set of 112 mutations coming from 17 complexes sharing less than 30% sequence identity with those seen during training (Fig. 4). DLA-mutation’s baseline version, exploiting only structural information, still outperforms all other methods except BindProfX (Supplementary Fig. S8).

In absolute terms, DLA-mutation’s performance is moderate in these two experiments (PCC below 0.5). In the first case, we may interpret the difficulty faced for generalizing from an older to a more recent version of SKEMPI as reflecting differences in the way these two versions were compiled and in the quality of their data (see Section 5). The balance of mutation types in the train and test sets may also play an important role. While the substitutions to alanine represent less than half of the mutations in the train set (SKEMPI v1.0), they amount to about 75% in the test set (SKEMPI v2.0). The substitutions to alanine also represent a large proportion (83 out of 112) of the mutations in the second experiment’s test set.

### 4.5 DLA-mutation performs better on core and rim and is robust to size and sequence identity changes

**Figure 5.**
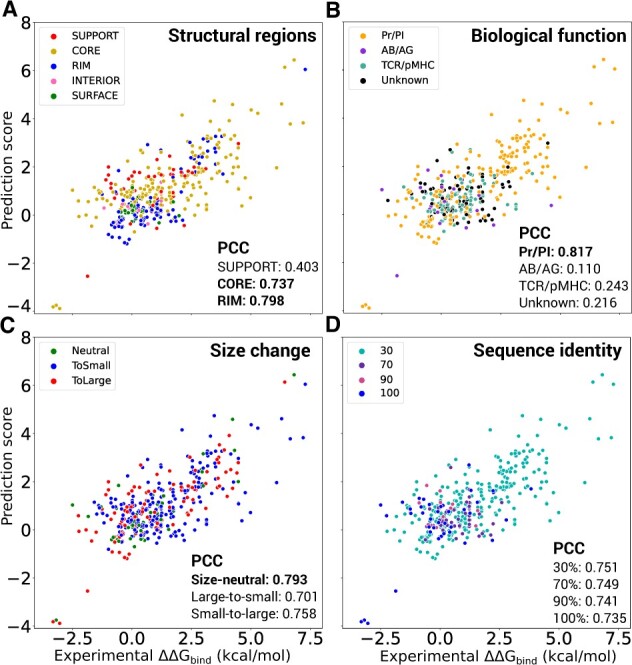
Influence of residue-based and complex-based properties on DLA-mutation accuracy. The predicted and experimental values are reported for 391 mutations coming from 32 complexes not seen during training (randomly selected from S2003 dataset). We used weight fine-tuning and all auxiliary features. The overall PCC is 0.720 (Table 1). The dots are colored with respect to the SR where the mutated residue lies (A), the complex’s biological function (B), the amino acid size change upon mutation (C), and the minimum sequence identity shared with any training complex (D). We calculated the change of amino acid size as a volume difference (*δV*) between wild-type and mutant following (Harpaz et al. 1994). A mutation was classified as size-neutral if $|\delta V|<10{Å}^{3}$, as small-to-large if $\delta V>10{Å}^{3}$, and as large-to-small if $\delta V<-10{Å}^{3}$.

The location of a mutation in a protein interface might be a relevant indicator for the confidence in the estimation. We investigated this issue by describing an interface as three concentric layers of residues, the support (internal layer), the core (the second layer), and the rim (the third and most external layer) (Levy 2010). DLA-mutation better deals with mutations taking place in the core and rim, with PCCs as high as 0.737 and 0.798, respectively (Fig. 5A, compare gold and blue dots with red dots). The mutations in the core are also the most frequent ones. These results may reflect the more substantial mutation-induced conformational variations in the core and rim, compared with the support, which are captured by backrub protocol. Very few mutations are located outside of the interface and the associated range of experimental $\Delta \Delta G_{\text{Bind}}$ values is very narrow, making it difficult to distinguish them (Fig. 5A, pink and green dots). In addition, the prediction accuracy seems to depend on the function of the complex, with the protease-inhibitor class displaying the highest number of complexes and the highest accuracy (Fig. 5B). However, this observation may be interpreted in the light of the nature of the substitutions. The unbalance of substitutions to alanine we observed between train and test sets above can also be observed between the different functional classes. Indeed, the protease–inhibitor complexes display a wide variety of substitutions, while the other classes mostly display substitutions to alanine. More precisely, more than 95% of the mutations of protease-inhibitor class comprise substitutions to non-alanine amino acids. In contrast, more than 84% of mutations for other classes, particularly for T-cell receptor—major histocompatibility complexes, are substitutions to alanine. This is due to the unbalanced distribution of mutation types in the SKEMPI databases. Overall the predictions are more accurate when the mutant amino acid is not alanine (PCC of 0.790 versus 0.34). The amino acid size change itself is not a determining factor (Fig. 5C). DLA-mutation performs consistently well on small-to-large, large-to-small, and size-neutral substitutions, with a slight preference for the latter (PCC = 0.793). The predictions are robust to variations in the sequence identity between the test and train complexes (Fig. 5D). Finally, we found that DLA-mutation had difficulties in accurately estimating the effects of substitutions to alanine. However, this trend is not homogeneous across complexes, as illustrated by the good predictions obtained for complexes 3M62, 1CHO, and 1JCK (Supplementary Fig. S9).

### 4.6 Predicting residue- and interface-based properties

To evaluate the embedding vectors computed by the pre-trained ssDLA, we tested whether they could be mapped to per-residue and per-interaction physicochemical and functional properties. To do so, we added a two-layer fully connected network on top of ssDLA’s architecture (Fig. 1D), and we trained it to perform two downstream tasks (see Section 2) The first task consisted in assigning amino acid physicochemical classes to the input cubes. The amino acid classification we chose previously proved relevant for predicting the functional impact of mutations (Laine et al. 2019). It distinguishes the aromatic amino acids (ARO: F, W, Y, H), the hydroxyl-containing ones plus alanine (CAST: C, A, S, T), the aliphatic hydrophobic ones (PHOB: I, L, M, V), the positively charged ones (POS: K, R), the polar and negatively charged ones (POL-N: N, Q, D, E), glycine (GLY), and proline (PRO) (Supplementary Table S4). The per-class tendencies are consistent with those observed for the pre-training task (Fig. 2, compare the two panels). Specifically, the best performances are observed for the aromatic (ARO) and positively charged (POS) classes, with more than 70% recall, while the CAST class is the most difficult to identify. Conformational sampling influences the results. We observed improved performances when dealing with 3D models compared with experimental structures (Supplementary Fig. S10). We may hypothesize that the backbone rearrangements and side-chain repacking performed by the backrub protocol lead to a better fit between the central amino acid and its environment (compare panels A and B). Averaging the embedding vectors over 30 models allows extracting with an even higher precision the intrinsic properties of the central amino acid (compare panels B and C). The second task was to predict the function of a protein–protein interaction. The embedding vectors proved useful to distinguish the protease–inhibitor assemblies (recall = 83.33%) from the two other functional classes (Supplementary Fig. S11). The classifier tends to confuse the antibody-antigens with T-cell receptor-major histocompatibility complexes. This behavior is expected, owing to the structural similarity shared between T-cell receptors and antibodies.

## 5 Discussion

Knowledge acquisition and transfer from protein–protein interfaces with deep learning approaches are useful to address the fundamental questions about protein–protein interactions. Our approach leverages the non-redundant set of experimentally resolved protein complex structures to assess the impact of mutations on protein–protein binding affinity, among other applications. Compared with other state-of-the-art predictors, DLA-mutation generalizes better to unseen complexes.

Despite the improvement over the state-of-the-art, the DLA-mutation generalization capability from the first to the second version of SKEMPI remains limited. This result likely reflects differences in the protocols employed to produce, collect and manually curate the data between the older version, released in 2012, and the new one, released 7 years later. The experimental methods used for measuring the binding affinities are not reported in the first version; therefore the reliable entries cannot be selected. Moreover, various strict checks with up-to-date references were applied for the second version to ensure its quality. In general, $\Delta \Delta G_{\text{Bind}}$ measurements may contain errors, e.g., coming from systematic bias or experimental uncertainty. In SKEMPI v2.0, we observed that for some mutations, distinct values of mutant binding affinity were measured by different laboratories or using different experimental techniques (Jankauskaitė et al. 2019).

Future work will more thoroughly investigate the contribution of conformational sampling and the quality of the $\Delta \Delta G_{\text{Bind}}$ prediction. Alleviating the need for precise models and substantial sampling would improve the scalability of the approach. Expanding the train set for ssDLA could also help the model learn residue-specific pattern variations and improve the performance. DLA-Mutation is designed to measure the changes of binding affinity caused by single-point mutations. The model could be used as is to predict the effects of multiple mutations, but only by predicting the effect of single mutations and then summing them up, which would be a crude approximation. Future improvements will aim at generalizing the DLA framework to properly deal with multiple-point mutations. Another direction for improvement concerns the treatment of substitutions to alanine. DLA-mutation generalization capability is also limited for this type of mutation. Overall, the results suggest that DLA-mutation would benefit from a simplified version of the architecture for performing computational alanine scans, which relies only on X-ray crystal structure. Combining DLA-mutation with alanine scans performed on the wild-type complex would open the way to systematically assess mutational outcomes on protein–protein interactions at a proteome-wide scale.

## Supplementary Material

btad231_Supplementary_Data

## Data Availability

The data underlying this article are available in the article, in its online supplementary material, and in the repository http://gitlab.lcqb.upmc.fr/DLA/DLA.git.
