# Supplementary material for: Deep Local Analysis deconstructs protein–protein interfaces and accurately estimates binding affinity changes upon mutation
Source: Bioinformatics. 2023 Jun 30;39(Suppl 1):i544–52. doi: 10.1093/bioinformatics/btad231 (PMC10311296; doi:10.1093/bioinformatics/btad231)
Supplement: btad231_Supplementary_Data [file btad231_supplementary_data.pdf]

Supplementary Information

Deep Local Analysis deconstructs protein - protein interfaces and accurately estimates binding affinity changes upon mutation

by Yasser Mohseni Behbahani, Elodie Laine and Alessandra Carbone

Definition of the interfacial residues

We define interfacial residues as those displaying a change in solvent accessibility between the free (isolated) protein and the complex (Levy, 2010). We used NACCESS (Hubbard and Thornton, 1993) with a probe radius of 1.4Å to compute residue solvent accessibility.

Building the cubic volumetric map

To build the cubic volumetric map, the atomic coordinates of the input structure are first transformed to a density function (Pagès *et al.*, 2019). The density d at a point $\vec{v}$ is computed as

$$\mathbf{d}(\vec{v}) = \sum_{i \leq N_{\text{atoms}}} \exp\left[-\left(\frac{\vec{v}-\vec{a_i}}{\sigma}\right)^2\right]\mathbf{t_i}, \quad \text{(S 1)}$$

where $\vec{a_i}$ is the position of the ith atom, σ is the width of the Gaussian kernel set to 1Å, and t_i is a vector of 167 channels corresponding to residue-specific atom types, or 4 channels corresponding to the four amino acid-independent chemical elements (O, C, N and S) (see (Pagès *et al.*, 2019) for a detailed list). The hydrogen atoms are discarded. Then, the density is projected on a 3D grid comprising $24 \times 24 \times 24$ voxels of side 0.8Å. The map is oriented by defining a local frame based on the common chemical scaffold of amino acid residues in proteins (Pagès *et al.*, 2019). More precisely, for the nth residue, the ($\vec{x}$, $\vec{y}$, $\vec{z}$) directions and the origin of the cube are defined by the position of the atom N_n, and the directions of C_{n-1} and $C\alpha_n$ with respect to N_n. The X-axis is parallel to the vector pointing from C_{n-1} to N_n. The Y-axis, perpendicular to the X-axis, is defined in such a way that $C\alpha_n$ lies in the half-plane Oxy with $y > 0$. The Z-axis is defined as the vector product $X \times Y$. The origin of the cube is determined in such a way that N_n is located at position (6.1Å, 6.6Å, 9.6Å). This choice ensures that all the atoms of the central residue fit in the cube. More details can be found in (Pagès *et al.*, 2019). This representation is invariant to the global orientation of the structure while preserving information about the atoms and residues relative orientations.

Auxiliary features

For predicting $\Delta\Delta G_{bind}$, we combined the embedding vectors of the volumetric maps with five pre-computed auxiliary features (**Fig. 1**C), among which four describe the wild-type residue:

- a one-hot vector encoding the protein structural region to which it belongs, either the interior (INT), the surface (SUR), or, if it is part of the interface, the support (S or SUP), the core (C or COR), or the rim (R or RIM), as defined in (Levy, 2010). We directly took the annotations available in the SKEMPI database (Jankauskaitė *et al.*, 2019) (see below for a description of the database). We previously demonstrated the usefulness of the S-C-R classification for predicting and analysing protein interfaces with other macromolecules (protein, DNA/RNA) (Mohseni Behbahani *et al.*, 2022; Corsi *et al.*, 2020; Raucci *et al.*, 2018; Laine and Carbone, 2015).
- its physico-chemical properties (PC, a float value) to be found at interfaces, scaled between 0 and 1 (Negi and Braun, 2007).
- its circular variance (CV, a float value) (Mezei, 2003; Ceres *et al.*, 2012) with a sphere radius of 12 Å on the protein structure. For each protein atom, CV measures the density of protein atoms around it within a sphere. The CV of a given residue is obtained by averaging values over its atoms and indicates its degree of burial in the protein. CV values range from 0 to 1 and protruding residues have a value close to 0.
- its conservation level T_{jet} (a float value) determined by the Joint Evolutionary Trees (JET) method (Engelen *et al.*, 2009). JET estimates evolutionary conservation by explicitly accounting for the topology of the phylogenetic tree relating the query protein to its homologs.

We used the JET2 package (Laine and Carbone, 2015) to compute PC, CV and T_{jet}. We previously showed the usefulness of these properties for detecting protein-protein interfaces and inferring their functions (Laine and Carbone, 2015). The fifth feature is specific of the mutation, that is

- a numerical score (a float value) estimating the functional impact of point mutations from multiple sequence alignments computed for single (monomeric) proteins by GEMME (Laine *et al.*, 2019). To do this estimation, GEMME combines the conservation levels T_{jet} with amino acid frequencies and the minimum evolutionary distance between the protein sequence and a homologous protein presenting the mutation.

We built the input multiple sequence alignments for JET and GEMME by performing five iterations of the profile HMM homology search tool Jackhmmer (Eddy, 2011) against the UniRef100 database of non-redundant proteins (Suzek *et al.*, 2015) using the EVcouplings framework (Hopf *et al.*, 2019). We used the default bitscore threshold of 0.5 bit per residue.

Experimental values for $\Delta\Delta G_{bind}$

We used SKEMPI v2.0 (Jankauskaitė *et al.*, 2019), the most complete source for experimentally measured binding affinities of wild-type and mutated protein complexes. It includes the smaller databases AB-Bind, PROXiMATE, and dbMPIKT (Geng *et al.*, 2019a). In total, it reports measurements for over 7 000 single and multiple point mutations coming from 345 protein complexes, including antibody-antigen (AB/AG) and protease-inhibitor (Pr/PI) assemblies, and assemblies formed between major histocompatibility complex proteins and T-cell receptors (pMHC-TCR). For each entry, corresponding to a single or multiple mutation, the database provides the PDB structure of the wild-type complex, the names of the partners, the binding affinities of the wild-type and mutated complexes, some related experimental measurements, details about the experimental method and conditions, and the structural region of the mutation site(s), either INT, SUR, SUP, COR or RIM (Levy, 2010). The mutations happening in the interface (SUP, COR, RIM), in particular in the core (COR), induce bigger changes in binding affinity than the ones located in the non-interacting surface (SUR) or the interior (INT) of the protein (**Fig. S 12**). Overall, we observed a tendency for the mutations to be deleterious rather than beneficial. The most impactful single-point mutation is located in the complex 1CHO with $\Delta\Delta G_{bind} = 8.802$ kcal/mol. This rich body of annotations helps us to analyze our results and identify the weak and strong points of DLA-mutation by evaluating its performance with respect to different classifications.

We restricted our experiments to the entries for which the binding affinity of the wild-type and mutant complexes were determined using a reliable experimental method, namely ITC, SPR, FL, or SP, as done in (Vangone and Bonvin, 2015). This first filtering step led to 4 974 entries associated with 255 protein complexes. We retained 4 634 entries from 245 complexes by excluding mutation entries with ambiguous free energy or without energy change. We then focused only on 3 393 single-mutation entries coming from 222 complexes. After removing duplicated entries (a protein complex with the same mutations), we remained with 2 975 mutations. We finally randomly selected a subset of 2 003 mutations associated with 142 complexes due to the computational cost of Backrub modeling. We call this subset *S2003*.

Protein-protein complex 3D structures

We created two databases of protein-protein complex 3D structures, namely *PDBInter* and *S2003-3D*, for training and validation purposes. PDBInter was curated from the Protein Data Bank (PDB) (Berman *et al.*, 2002) and thus contains only experimental structures. S2003-3D was generated using the "backrub" protocol implemented in Rosetta (Smith and Kortemme, 2008) and thus contains only 3D models.

PDBInter. We downloaded all PDB biological assemblies (June 2020 release) from the FTP archive `rsync.wwpdb.org::ftp/data/biounit`rsync.wwpdb.org::ftp/data/biounit. We discarded the entries with more than 100 chains or with a resolution lower than 5Å. We also removed the protein chains smaller than 20 residues or with more than 20% of unknown residues. We then redundancy-reduced the resulting dataset using annotations from the SCOPe database (Fox *et al.*, 2014; Chandonia *et al.*, 2022). The 5 055 protein complex structures that were finally retained do not share any family level similarity between them according to the SCOPe hierarchy.

S2003-3D. We generated 3D models with a high level of precision using the Rosetta backrub protocol for the wild-type and mutated complexes from S2003 and explicitly accounted for the conformational variability. We followed a modeling protocol similar to that reported in (Barlow *et al.*, 2018). It relies on the backrub method (Smith and Kortemme, 2008) for sampling side chain and backbone conformational changes. Our goal was to accurately mimic and explore the fluctuations around a native state. The protocol unfolds in two optimization steps carried out on the side chains and the backbone (**Fig. S 6**):

1. for the backbone and the side chains, it applies quasi-Newton minimization for continuous optimization of torsion angles: Φ, Ψ, χ_1, χ_2, χ_3, etc.
2. for the side chains only, it performs Monte Carlo simulation with the backbone-based side-chain rotamer library of Dunbrack (Shapovalov and Dunbrack, 2011) for discrete combinatorial rotamer optimization, also known as repacking.

Split complexes based on sequence identity

To split train and test sets based on complex sequence identity, we directly exploited the clusters of sequence identity available from the PDB `https://www.rcsb.org/docs/programmatic-access/file-download-services#sequence-clusters-data`. Two protein complex have the same sequence identity if all their chains share the same clusters.

Evaluation of ssDLA on the validation set from PDBInter

To visualise the performance of the model, we generated logos from pseudo alignments of 20 columns corresponding to the 20 amino acids. In the column corresponding to the amino acid a_i, the frequency of occurrence of each amino acid a_j corresponds to the propensity of ssDLA to predict a_j when the true central residue of the input cube is a_i. Note that the propensity is computed by counting the number of times a_j has maximum probability score among the 20 candidate amino acids. If some amino acid was never predicted, we simply put a gap character.

Different experimental setups for the supervised prediction of $\Delta\Delta G_{bind}$

We experimented different setups of supervised learning by using different combinations of auxiliary features and different initialisation schemes for the network weights. In the basic set up, the only auxiliary feature we used was the structural region of the wild-type residue (SR). We previously showed that this information significantly contributes to the performance of the DLA framework (Mohseni Behbahani *et al.*, 2022). On top of that, we also considered evolutionary information, by using the *GEMME* scores (*SR-GEMME*) or the T_{jet} conservation levels (*SR-Tjet*). In its most complete form, DLA-mutation combines SR, *GEMME* scores, T_{jet}, and descriptors of the buriedness (CV) and physico-chemical properties (PC) of the wild-type residue (*All*). For the network weights, we either started from the weights of the pre-trained ssDLA (*fine tuning*) or randomly initialised them. For each mutation, DLA-Mutation considers 30 pairs of cubes extracted from the mutation site of the associated 30 backrub models. The predicted $\Delta\Delta G_{bind}$ is an average over all 30 models.

Training and evaluation of downstream tasks: prediction of residue- and interface-level properties

We generated embedding vectors (e_k) for all the cubes representing a given input interface. For training purposes, we redundancy-reduced the set of 142 complexes from *S2003* based on a 30% sequence identity cutoff. We then performed a 50/50 split at the cluster level. This resulted into 85 train and 57 test complexes for the first, residue-based, task. As training and testing samples, we considered:

- either all interfacial residues (4710 residues for train and 3397 residues for test) extracted from the X-ray crystal structures of *S2003*;
- or only the residues belonging to the positions with mutation from S2003 (1700 residues for train and 303 residues for test) extracted from the wild-type backrub models of *S2003-3D*. We performed two experiments here: (i) pick up one backrub model at random (out of 30) to generate the input cubes, (ii) average the embedding vectors computed for a given interfacial residue over the 30 backrub models.

For the second, interface-based task, due to missing annotations, we used only 22 train and 52 test complexes. We computed the average embedding vector over all interfacial residues before giving it to the classifier. In addition, we focused only on the X-ray crystal structures. In both tasks, the number of epochs depended on the size of train set and the learning rate (0.00001). We stopped the training when the validation loss converged to a steady value (**Fig. S 13**).

Table S 1. Benchmark datasets of changes of binding affinity upon mutation

Name	Number of complexes	Number of mutations	Type of point mutations	Source Database
ZEMu (Dourado and Flores, 2016)	65	1240	single+multiple	SKEMPI1
S1102 (Geng *et al.*, 2019b)	57	1102	single	SKEMPI1
S487 (Geng *et al.*, 2019b)	56	487	single	SKEMPI2
S645 (Pires and Ascher, 2016)	29	645	single	AB-Bind
S787 (Wang *et al.*, 2020)	24	787	single	AB-Bind
S4947 (Wang *et al.*, 2020)	-	4947	single	SKEMPI2
S4169 (Rodrigues *et al.*, 2019)	319	4169	single	SKEMPI2
S8338† (Rodrigues *et al.*, 2019)	319	8338	single	SKEMPI2
S1721 (Rodrigues *et al.*, 2021)	147	1721	single+multiple	SKEMPI2
S1402 (Xiong *et al.*, 2017)	114	1402	single+multiple	SKEMPI1
S1131 (Xiong *et al.*, 2017)	114	1131	single	SKEMPI1
M1707 (Zhang *et al.*, 2020)	120	1707	multiple	SKEMPI2
S2003	142	2003	single	SKEMPI2

† S8338 is generated from S4169. It doubles the number of samples by assigning reverse mutation energy changes to the negative values of its original energy values in order to increase the robustness of the predictive method.

Table S 2. Different approaches for the prediction of changes of binding affinity upon mutation.

Approach	Type	Information	$\Delta\Delta G_{bind}$ directly	Train set	Test set	PCC	RMSE ($\frac{kcal}{mol}$)
FLEX (Barlow *et al.*, 2018)	Physics	Structure	-	-	ZEMu	0.63	-
BindProfX (Xiong *et al.*, 2017)	Physics+Statistics	Structure+Sequence	✓	-	S1402	0.691	-
					S1131	0.738	
iSEE (Geng *et al.*, 2019b)	ML	Structure+Sequence	✓	S1102	-	0.80*	1.41
				S1102	S487	0.25	1.32
mCSM-AB (Pires and Ascher, 2016)	ML	Structure	✓	S645	-	0.53	-
mCSM-PPI2 (Rodrigues *et al.*, 2019)	ML	Structure	✓	S4169	-	0.76*	1.19
				S8338	-	0.82*	1.18
mmCSM-PPI (Rodrigues *et al.*, 2021)	ML	Structure	✓	S1721	-	0.87*	1.41
				S1721†	S1721†	0.70	2.06
TopNetTree (Wang *et al.*, 2020)	ML	Structure	✓	S4947	-	0.82*	1.11
				S4169	-	0.79*	1.13
				S8338	-	0.85*	1.11
				S645	-	0.65*	1.57
				S4947	S787	0.53	1.45
				S1131	-	0.85*	-
Hom-ML-V2 (Liu *et al.*, 2022)	ML	Structure	✓	S4169	-	0.80*	1.06
				S645	-	0.58*	-
				S1131	-	0.857*	1.28
MuPIPR (Zhou *et al.*, 2020)	ML	Sequence	✓	S1102	-	0.85*	1.23
				S1400	-	0.88*	1.32
				S1102	S487	0.25	1.36
GraphPPI (Liu *et al.*, 2020)	ML	Structure	✓	S645	-	0.67*	-
				M1707	-	0.88*	-
				S645	-	0.53§	-
				M1707	-	0.76§	-
				S645	-	0.48¶	1.74
				M1707	-	0.73¶	2.26

* Mutation-based cross validation, in which a complex (or even the same mutation position of that complex) can be found in different folds. § Leave-one-complex-out cross validation. ¶ Leave-one-structure-out cross validation. † A subset of 1126 mutations used for training/CV and a subset of 595 mutations held out as non-redundant blind test at mutation level. ML: Machine learning, PCC: Pearson Correlation Coefficient, RMSE: Root Mean Squared Error.

Table S 3. Weights of amino acids classes in self-supervised learning

Amino acid	Weights	
	167 channels	4 channels
A	0.768	0.768
C	4.100	4.100
D	0.901	0.751
E	0.724	0.724
F	1.117	1.117
G	0.825	0.825
H	1.747	1.747
I	0.920	0.920
K	0.904	0.904
L	0.529	0.529
M	2.088	2.088
N	1.170	1.170
P	0.856	0.856
Q	1.182	1.182
R	0.717	0.717
S	0.885	0.885
T	0.920	0.920
V	0.817	0.817
W	2.897	2.897
Y	1.109	1.109

Table S 4. Seven classes of amino acids

Class name	Description	Amino acid(s)	Representative color
ARO	Aromatic	F, W, Y, H	Green
CAST	Hydroxyl-containing and Alanine	C, A, S, T	Black
PHOB	Aliphatic hydrophobic	I, L, M, V	Red
POS	Positively charged	K, R	Purple
POL-N	Polar and negatively charged	N, Q, D, E	Blue
GLY	Glycine	G	Gray
PRO	Proline	P	Orange

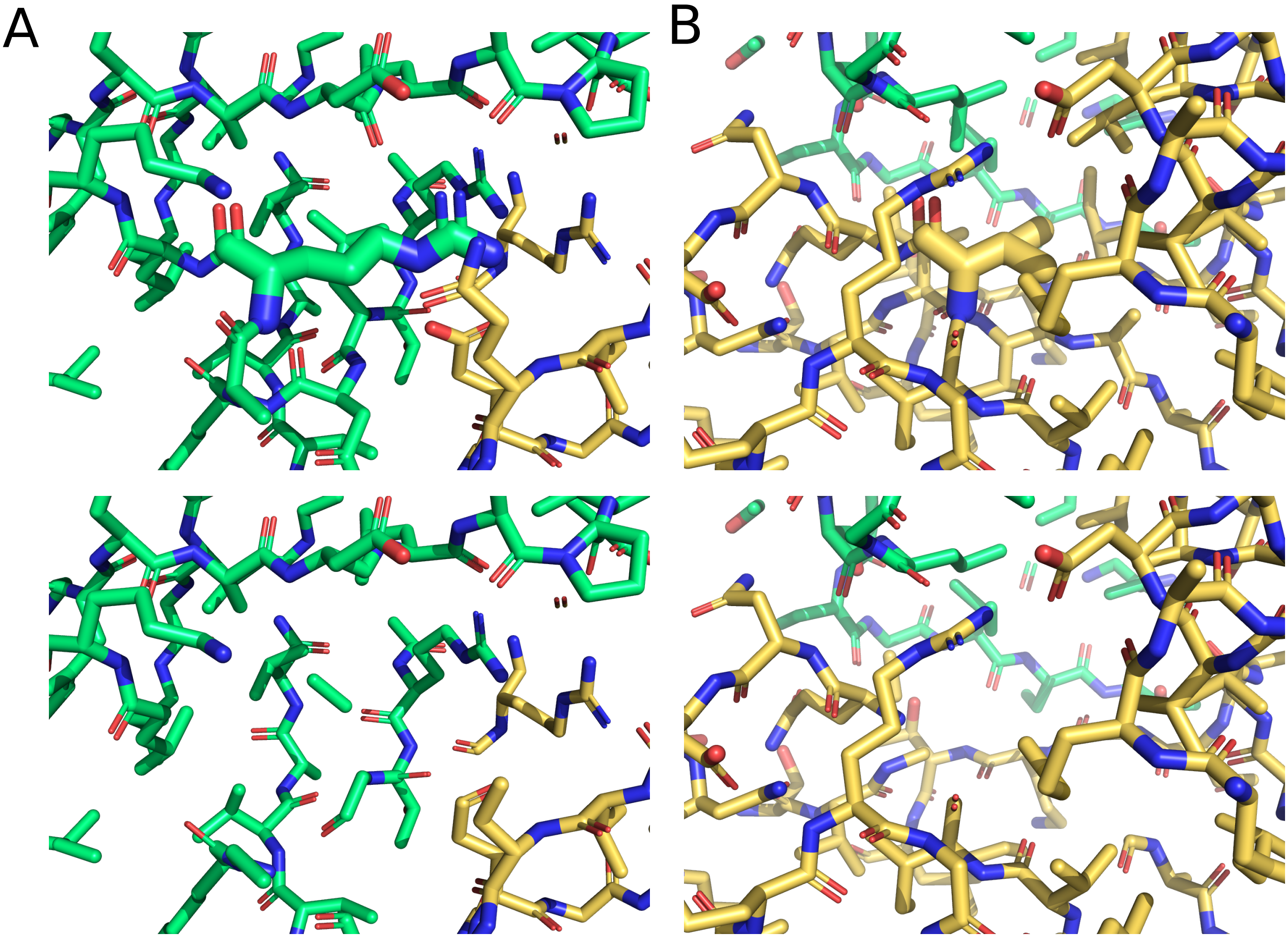

Fig. S 1. An example of masking with a sphere of 5 Å. Masking atoms inside a spherical volume of radius 5 Å randomly centered on the central interfacial residues (**A**) arginine or (**B**) isoleucine. Top is the intact local environment and bottom is the masked one. Both interfacial residues belong to the same interface.

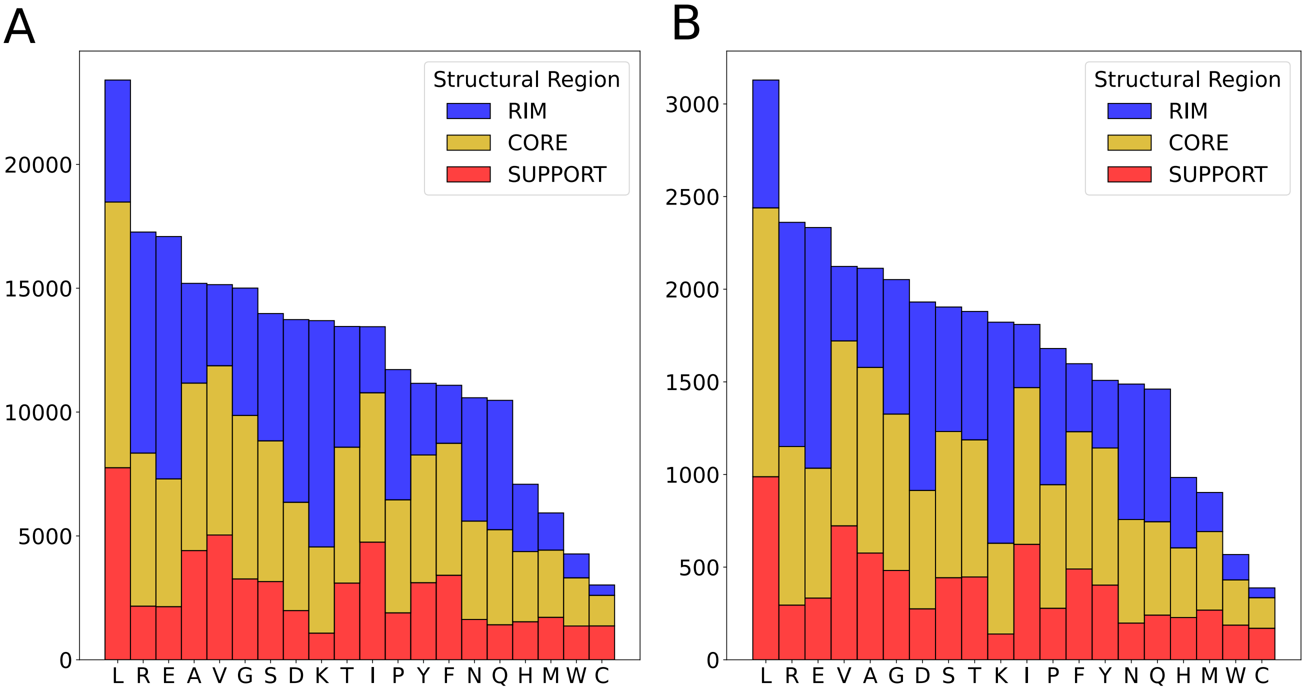

Fig. S 2. Frequency of interfacial amino acids in PDBInter. For each amino acid type, we report the number of times it appears in the core, the rim, or the support of the interface. **A** train set. **B.** Validation set.

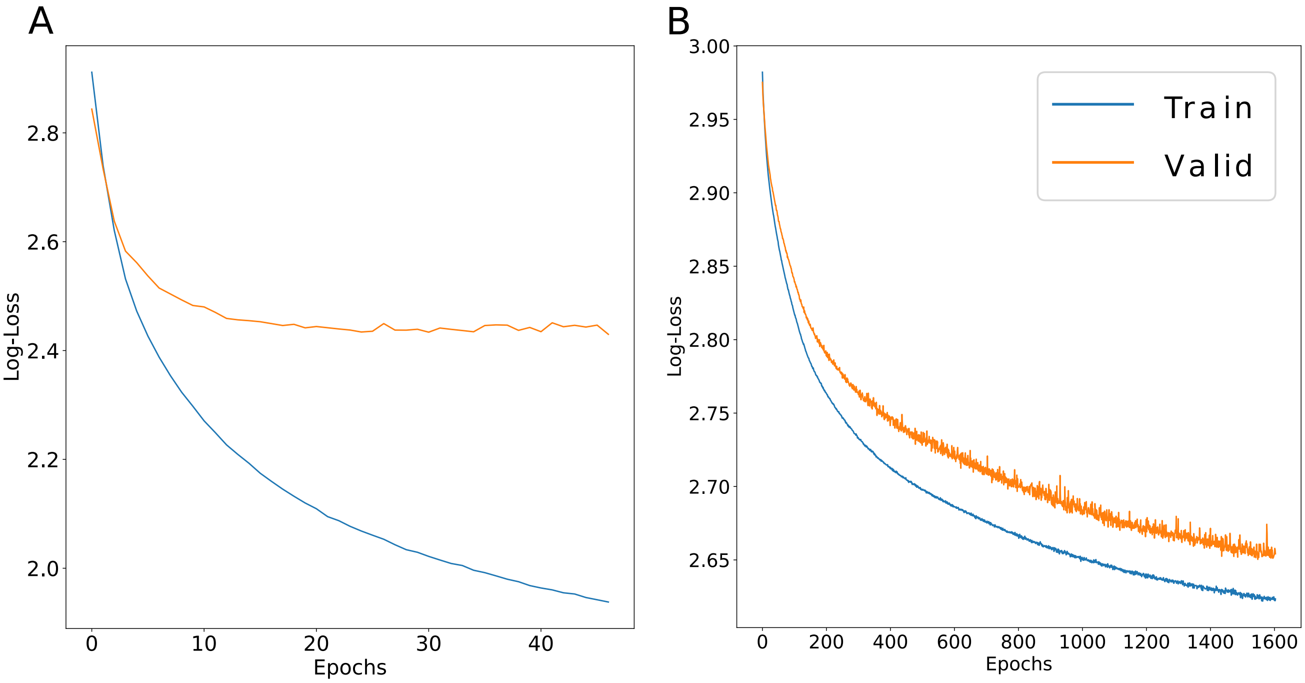

Fig. S 3. Train and validation loss curves of ssDLA. The x-axis is the number of epochs and the y-axis is the log-loss (categorical cross-entropy). (**A**) Default ssDLA model, where we used 167 channels corresponding to 167 amino acid-specific atom types (see (Pagès *et al.*, 2019) for a detailed list). (**B**) Simplified model where we considered only 4 atom types (C, N, O, S).

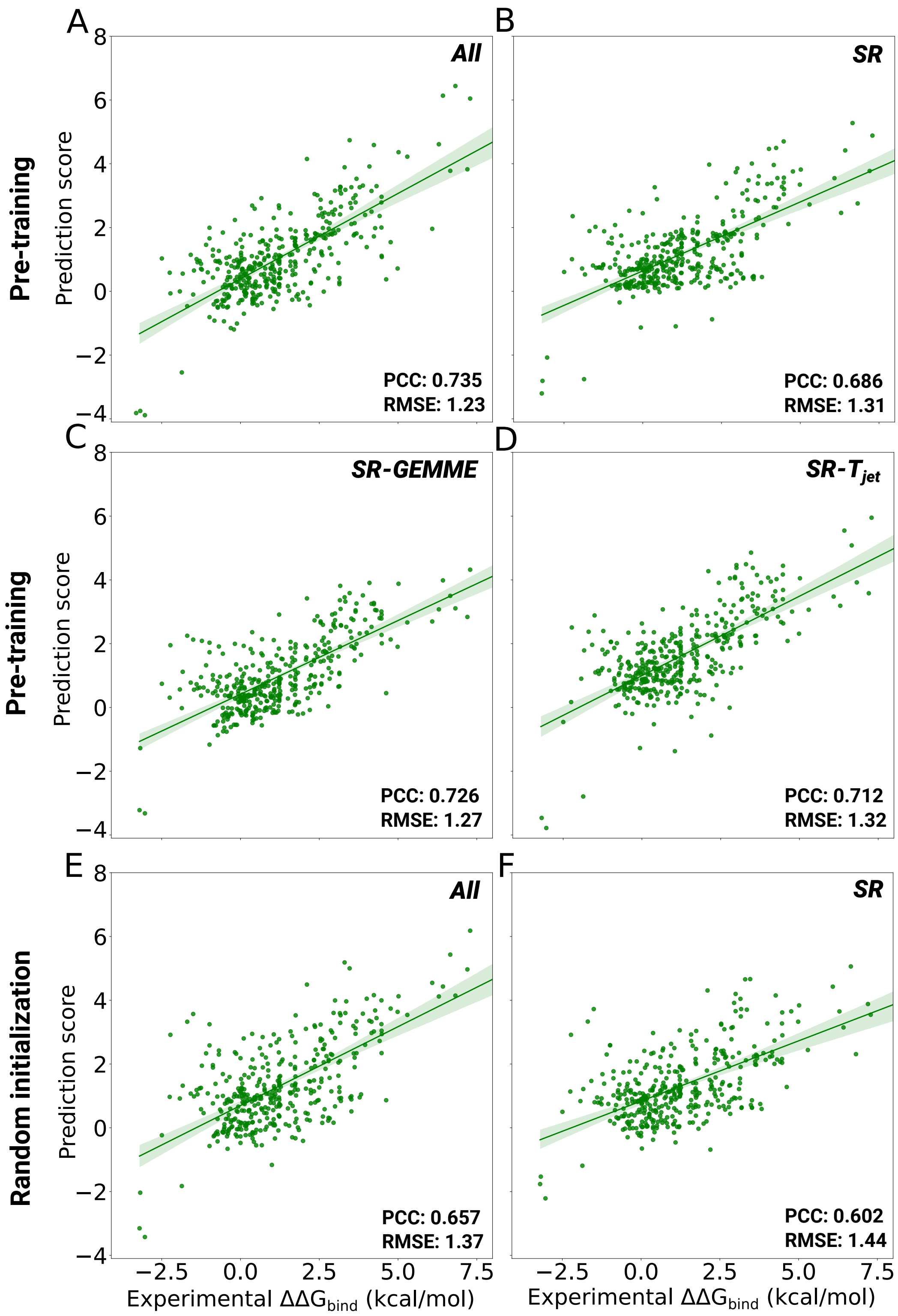

Fig. S 4. The predictive performance of six experimental setups on a test set of 391 mutations from 32 unseen protein complexes (randomly selected from S2003 dataset) with a complex-based train and test split. A-D. The training process fine-tunes the weights of the pre-trained model ssDLA and includes *All* (**A**), *SR* (**B**), *SR-GEMME* (**C**) or *SR-Tjet* (**D**) features. **E-F.** Training starts from randomly initialized weights with *All* (**E**) or *SR* (**F**) features.

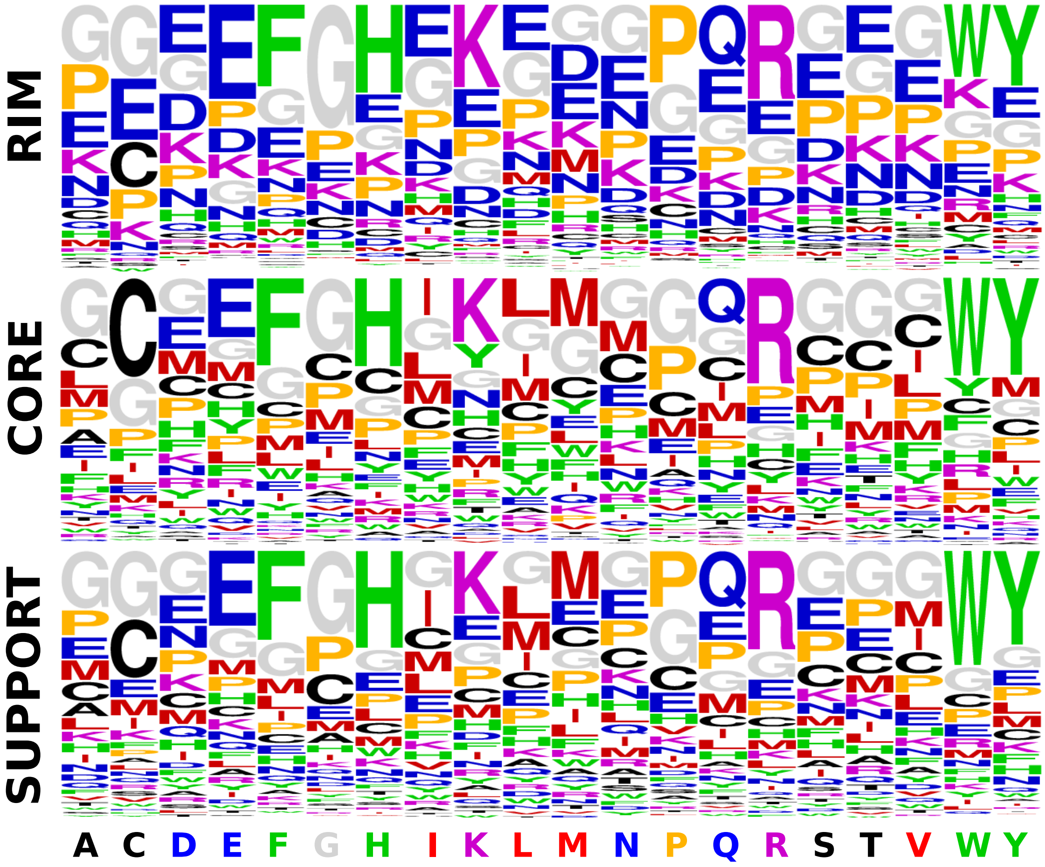

Fig. S 5. Performance of simplified ssDLA model with 4 channels. The predictive power of simplified ssDLA model where we considered only 4 atom types (C, N, O, S) is evaluated on the validation set of *PDBInter*. The three logos represent the propensities of each amino acid to be predicted (having maximum score in the output layer), depending on the true amino acid (x-axis) and on its structural region (see *Methods*). Amino acids are colored based on seven similarity classes: ARO (F, W, Y, H) in green, CAST (C, A, S, T) in black, PHOB (I, L, M, V) in red, POS (K, R) in purple, POL-N (N, Q, D, E) in blue, GLY (G) in gray and PRO (P) in orange (see *Methods*).

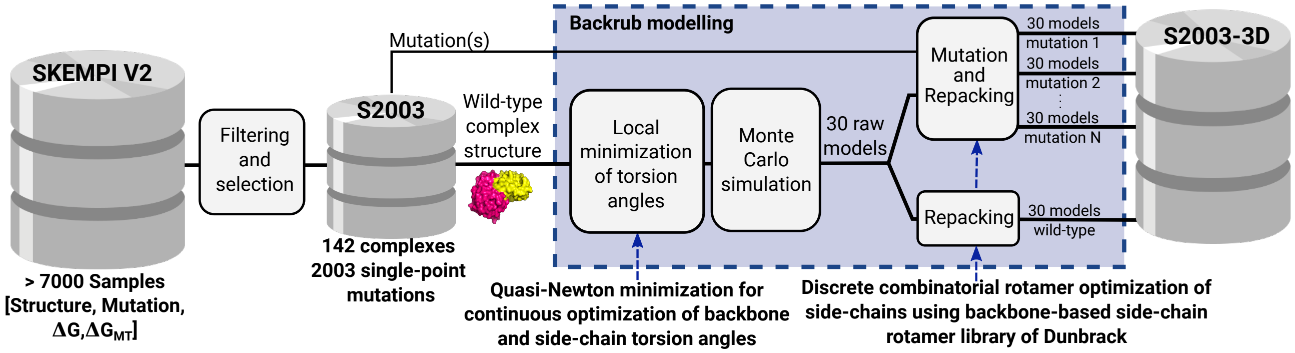

Fig. S 6. Pipeline for the generation of mutated complexes with backrub. After filtering the SKEMPI v2.0 database, we retained 2003 single point-mutations for 142 complexes (S2003). A wild-type structure undergoes a local minimisation of backbone and side-chain torsion angles followed by a Monte Carlo simulation step. We applied it to produce 30 models for each mutated structure and 30 for the wild-type. This process is followed by a repacking step applied to wild-type and mutation models. For the mutation positions at the interface of each model, we compute the associated cubic volumetric maps.

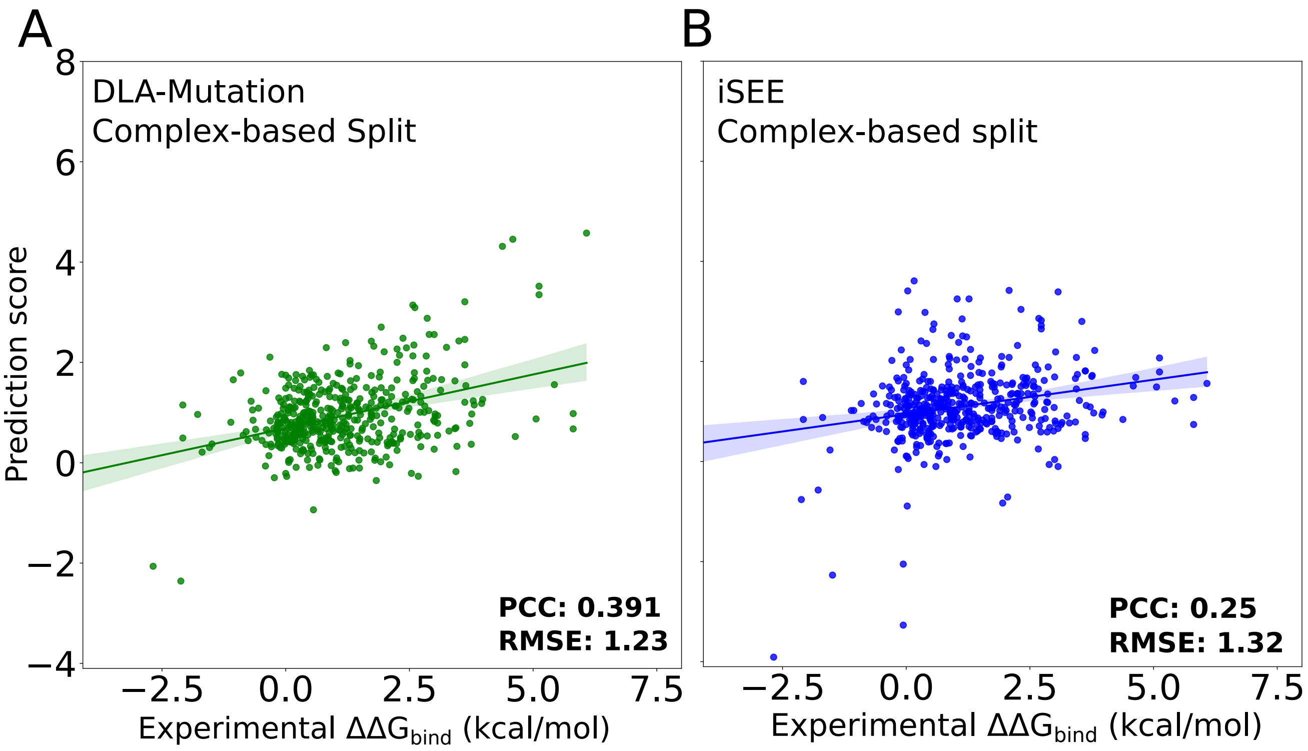

Fig. S 7. A comparison between the performance of DLA-Mutation (trained on only structural features; green) and iSEE (blue) on S487 dataset. The input 3D models and training and evaluation procedure were directly taken from (Geng *et al.*, 2019b). To train DLA-Mutation, we used fine-tuning of the weights and only structural information as auxiliary features (*SR*). **A.** DLA-Mutation **B.** iSEE.

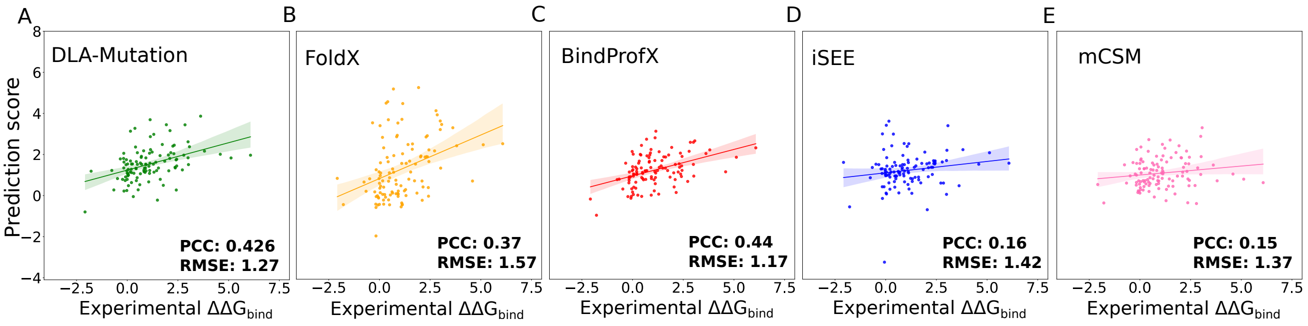

Fig. S 8. Comparison between DLA-Mutation (trained only on structural features) and other $\Delta\Delta G_{Bind}$ predictors. We report values for 112 mutations coming from 17 protein complexes not seen during the training or optimisation of any of the predictors. **A.** DLA-Mutation was trained on 945 mutations from S2003 coming from complexes sharing less than 30% sequence identity with those from this test set. To train DLA-Mutation, we used fine-tuning of the weights and only structural information as auxiliary features (*SR*). **B-E.** The scores reported for FoldX (B), BindProfX (C), iSEE (D) and mCSM (E) were taken directly from (Geng *et al.*, 2019b).

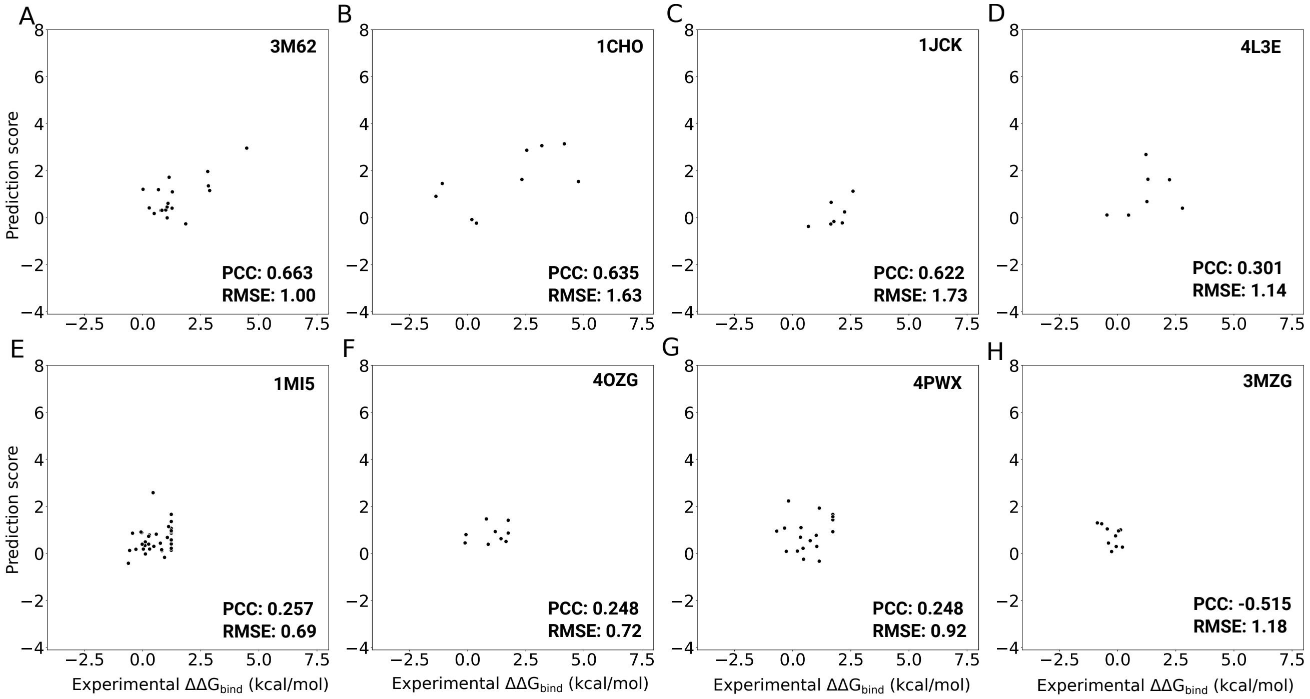

Fig. S 9. DLA-Mutation predictions for mutations to Alanine separated by different complexes. Predictions are obtained with the DLA-Mutation architecture with a fine-tuned pre-trained model and *All* auxiliary features. **A.** 3M62, **B.** 1CHO, **C.** 1JCK, **D.** 4L3E, **E.** 1MI5, **F.** 4OZG, **G.** 4PWX, **H.** 3MZG.

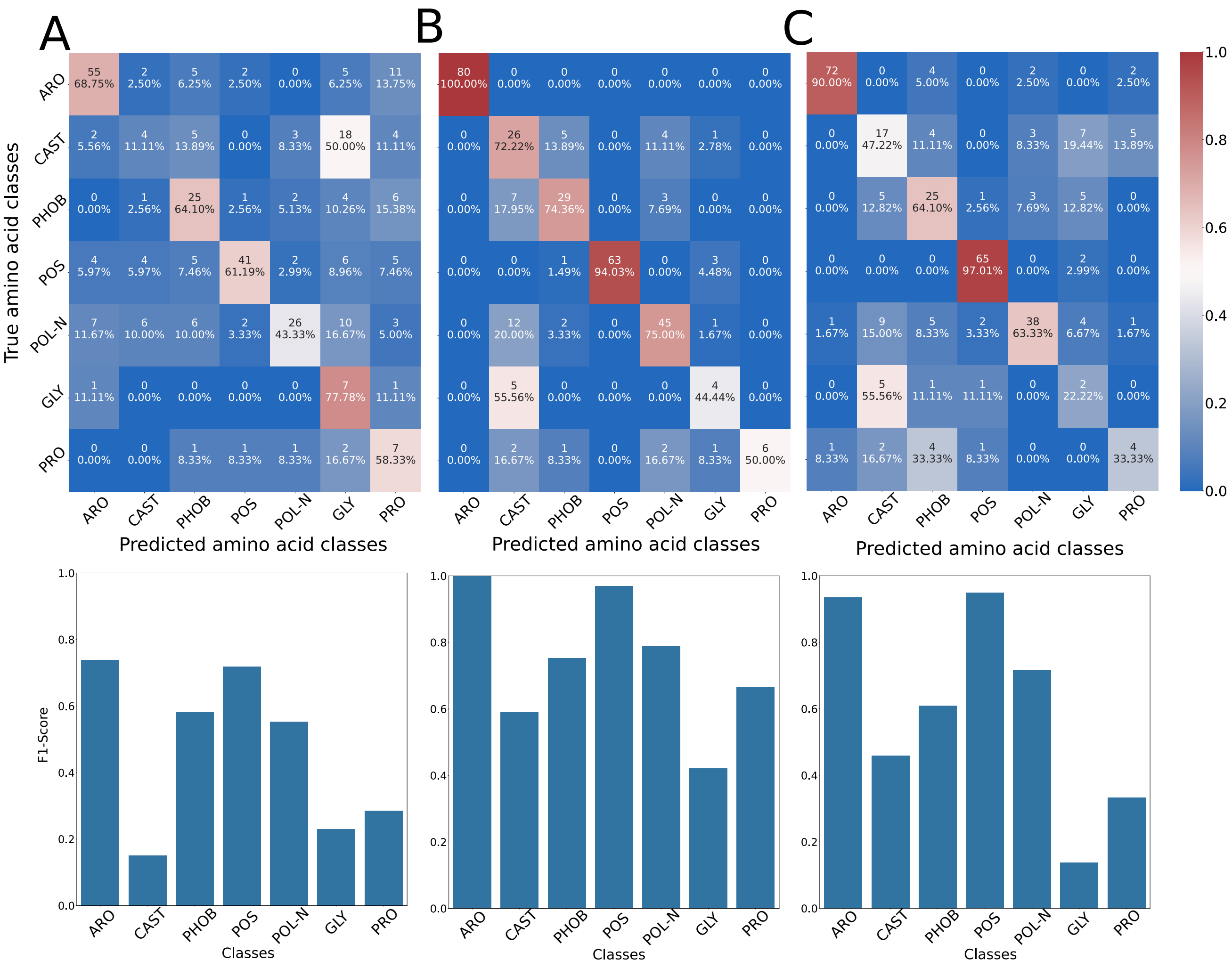

Fig. S 10. Prediction of the amino acid classes for the mutation sites using embedding vectors extracted by ssDLA. Train and test were performed on the subset of mutation sites of the X-ray crystal and wild-type backrub structures of S2003. **A.** The confusion matrix and per-class F1-scores for the embedding vectors of X-ray structures extracted by default ssDLA (167 channels). **B-C.** The confusion matrix and per-class F1-scores for the embedding vectors of wild-type backrubs extracted by default ssDLA with two aggregating schemes: averaging over backrub models (**B**) or choosing a single backrub model (**C**). In the confusion matrices the percentage values and the colors indicate recall.

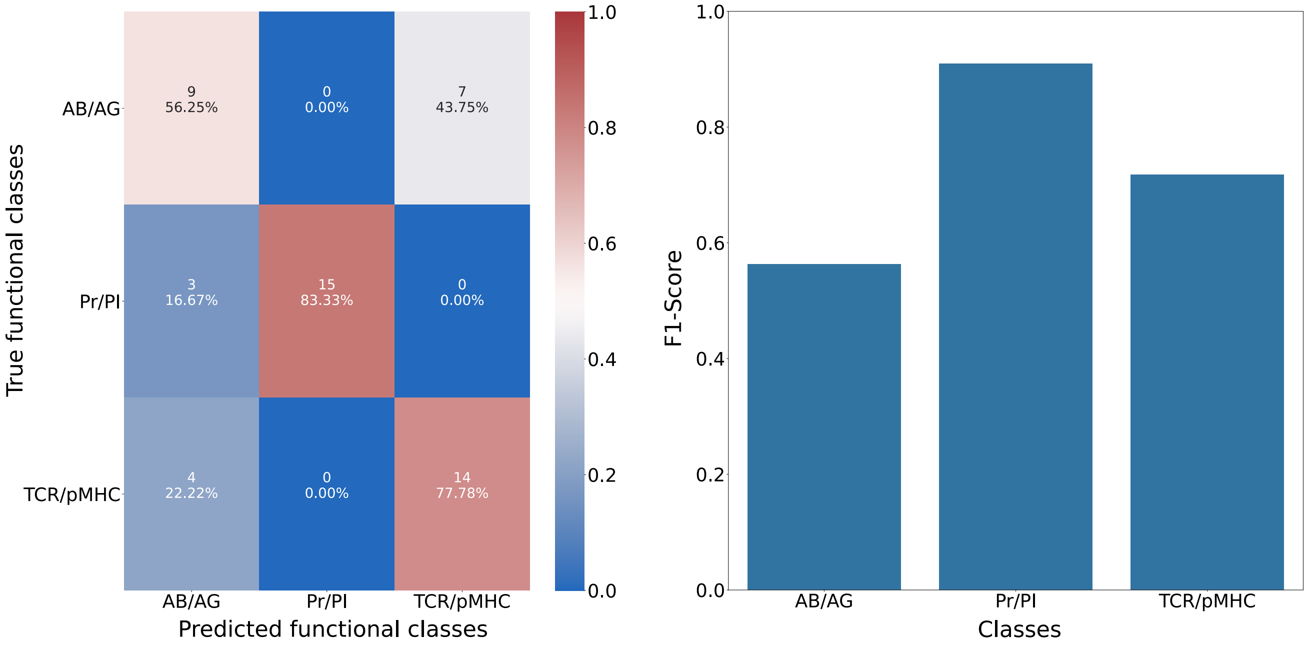

Fig. S 11. Prediction of the interaction functional classes using embedding vectors extracted by ssDLA. Train and test were performed on the X-ray crystal structures of S2003. **A-B.** The confusion matrix (**A**) and per-class F1-scores (**B**) for the embedding vectors extracted by default ssDLA (167 channels). In the confusion matrices the percentage values and the colors indicate recall. The aggregating scheme for each complex is the averaging of embedding vectors over is interfacial residues.

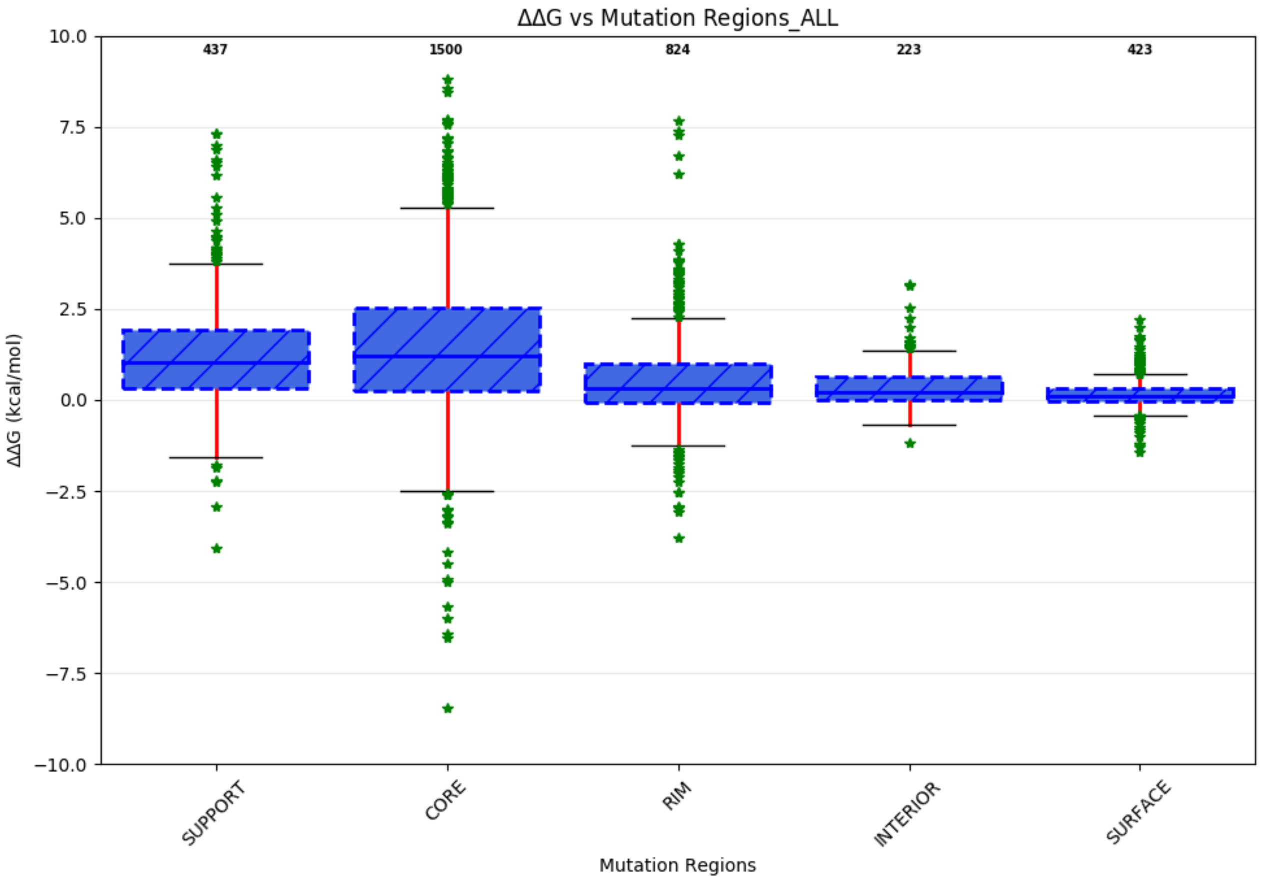

Fig. S 12. Distribution of $\Delta\Delta G_{bind}$ values in SKEMPI v2. We focus here only on the single point mutations. The different distributions correspond to the different protein structural regions: COR (1500 mutations), SUP (437 mutations), RIM (824 mutations), INT (223 mutations), and SUR (423 mutations).

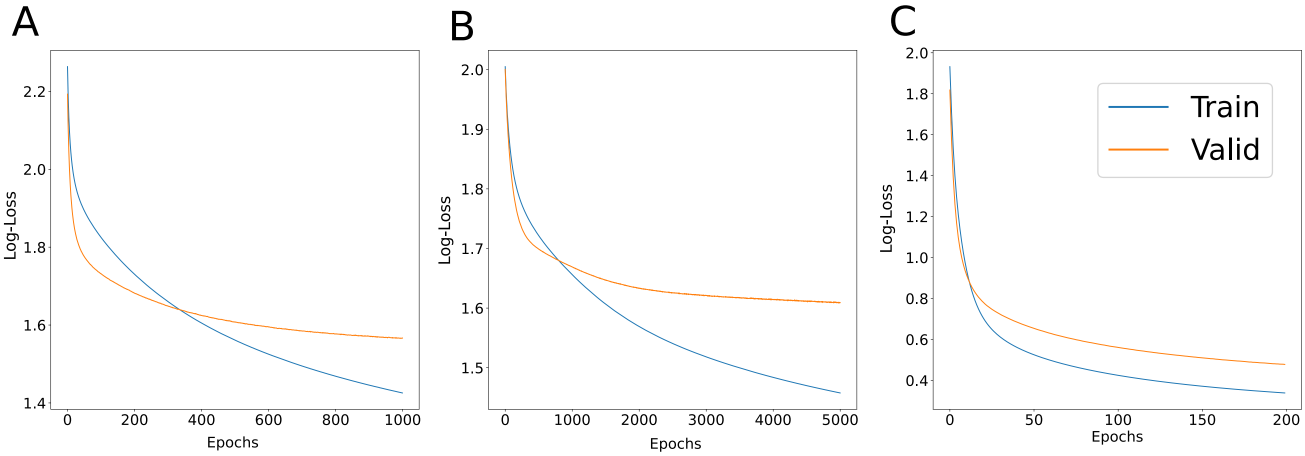

Fig. S 13. Train and validation loss curves of the downstream task: prediction of amino acid classes. x-axis is the epochs and the y-axis is the loss function (categorical cross-entropy). **A-B.** Embedding vectors extracted by default ssDLA model (167 channels, **A**) or by simplified model (4 channels, **B**) from X-ray crystal structures of S2003. **C.** Embedding vectors extracted by default ssDLA from wild-type backrub models of S2003.
